# Assessment of cardiac image quality from single-probe handheld ultrasound devices

**DOI:** 10.1093/ehjimp/qyag172

**Published:** 2026-10-06

**Authors:** Anna Grimaldo, Benjamin Koethe, Shane Williams, Eileen W Mai, Joseph Nguyen, Monica M Dehn, Lisa Small, Jennie An, Veronique Recile, Denise Daudelin, Ludovic Trinquart, David M Kent, Ayan R Patel, Michael Hughes, Benjamin S Wessler

**Affiliations:** Department of Medicine, Tufts Medical Center, Boston, MA, USA; Institute for Clinical Research and Health Policy Studies, Tufts Medical Center, Boston, MA, USA; Tufts Clinical and Translational Science Institute, Tufts Medical Center, Tufts University, Boston, MA, USA; Department of Computer Science, Tufts University, Medford, MA, USA; Cardiovascular Center, Tufts Medical Center, Boston, MA, USA; Cardiovascular Center, Tufts Medical Center, Boston, MA, USA; Cardiovascular Center, Tufts Medical Center, Boston, MA, USA; Predictive Analytics and Comparative Effectiveness (PACE) Center, Tufts Medical Center, Boston, MA, USA; Predictive Analytics and Comparative Effectiveness (PACE) Center, Tufts Medical Center, Boston, MA, USA; Cardiovascular Center, Tufts Medical Center, Boston, MA, USA; Institute for Clinical Research and Health Policy Studies, Tufts Medical Center, Boston, MA, USA; Tufts Clinical and Translational Science Institute, Tufts Medical Center, Tufts University, Boston, MA, USA; Institute for Clinical Research and Health Policy Studies, Tufts Medical Center, Boston, MA, USA; Tufts Clinical and Translational Science Institute, Tufts Medical Center, Tufts University, Boston, MA, USA; Predictive Analytics and Comparative Effectiveness (PACE) Center, Tufts Medical Center, Boston, MA, USA; Cardiovascular Center, Tufts Medical Center, Boston, MA, USA; Department of Computer Science, Tufts University, Medford, MA, USA; Tufts Clinical and Translational Science Institute, Tufts Medical Center, Tufts University, Boston, MA, USA; Cardiovascular Center, Tufts Medical Center, Boston, MA, USA; Predictive Analytics and Comparative Effectiveness (PACE) Center, Tufts Medical Center, Boston, MA, USA

**Keywords:** echocardiography, structural heart disease, handheld ultrasound, valve disease, cardiomyopathy

## Abstract

**Aims:**

This study aims to assess quality of cardiac ultrasound imaging from commercially available single-probe handheld devices.

**Methods and results:**

Cardiac imaging from single-probe handheld ultrasound devices was prospectively acquired by registered cardiac sonographers. Videos were graded on a scale of 1 (uninterpretable) to 5 (diagnostic-quality imaging) by 2 sonographers. The average image quality score for each cine loop was analysed. Interpretable imaging was defined as an average IQ score ≥ 2. Good image quality was defined as an average IQ score ≥ 4. Imaging from single-probe devices representing 518 videos from 206 patients was assessed. The mean age was 72 (SD 11.1) years. Agreement on image quality was moderate (weighted kappa 0.50, 95% CI 0.45–0.55). The correlation between sonographer quality grades was 0.60. The median average IQ score was 2.5 (1.5). 454 (88%) of the videos were interpretable (average IQ ≥ 2). At least one interpretable video was obtained for 187 (91%) patients. 62/518 (12%) of videos had good image quality (average score ≥ 4). There was a trend towards lower image quality with increasing BMI categories [16–25, median 3.0 (2.0, 3.5), 25–29, median 3.0 (2.0, 3.5), > 29 median 2.5 (2.0, 3.0), *P* < 0.001].

**Conclusion:**

Basic interpretable imaging from single-probe handheld ultrasound devices can be obtained for most patients under idealized conditions, suggesting possible use for enhanced cardiac disease detection. Since imaging from a significant minority of patients is uninterpretable and lower image quality is associated with higher BMI, rigorous assessment of image quality is essential prior to image interpretation.

## Graphical Abstract

**Graphical Abstract:**
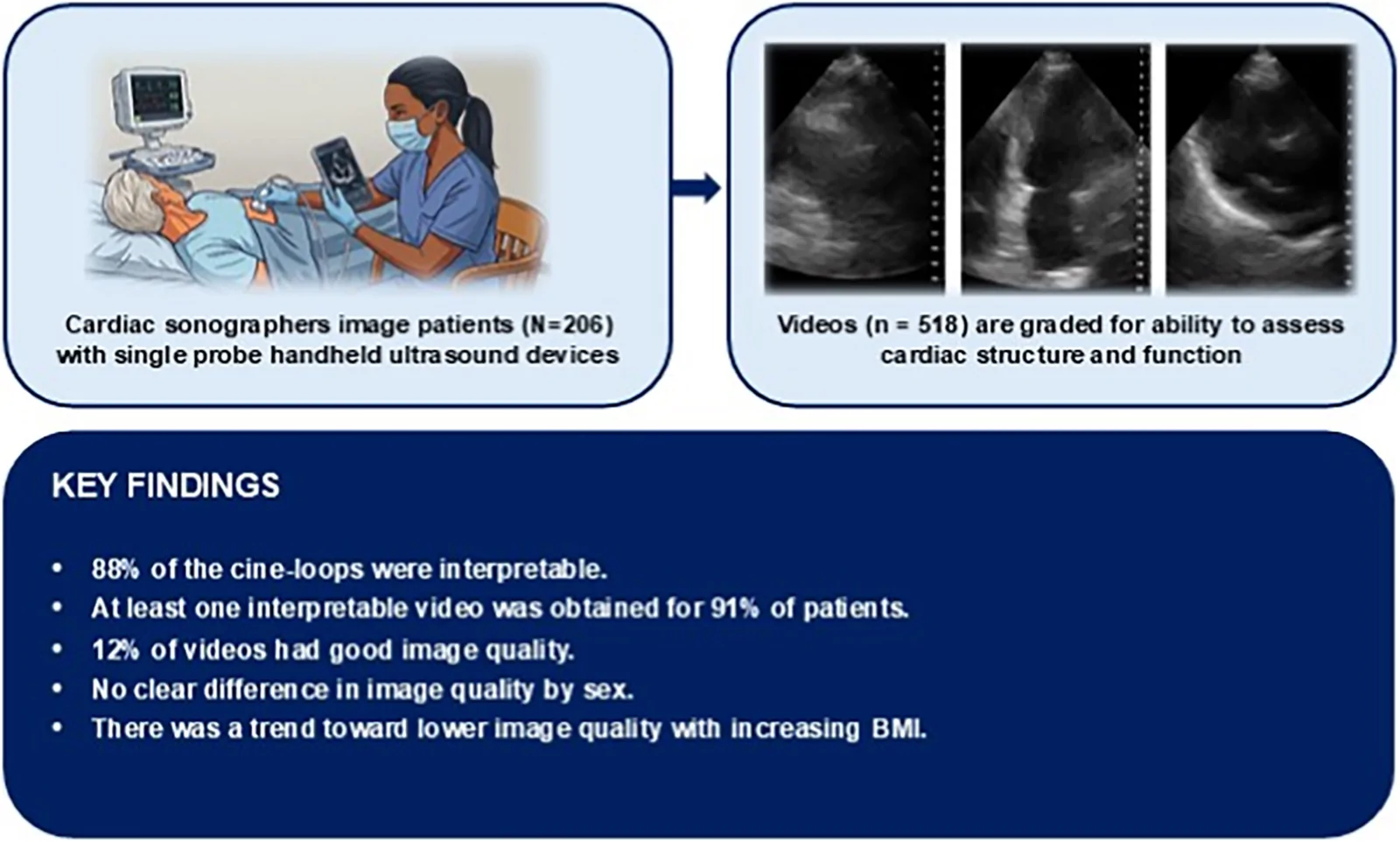

## Introduction

Cardiac auscultation is neither sensitive nor specific for detecting structural heart disease^1^ and handheld ultrasound is proposed as a method to improve disease detection.^2^ While substantial technological developments have led to improved device portability and substantially increased clinical use, there are major concerns about the quality of imaging from handheld ultrasound devices and how sub-optimal image quality might impact decision making.^3,4^

Single-probe handheld ultrasound devices allow imagers to switch between various array modes (linear, convex, phased) without the need to change out hardware. This advantage enables easier scanning of anatomic regions at various depths from the skin (cardiac, abdominal, musculoskeletal), and as a result, single-probe devices are particularly well suited to scanning by general, emergency and critical care practitioners (i.e. non-cardiologists). With an emerging body of evidence that structural heart disease is under-diagnosed and under-treated today^5^ there is interest in moving detection of structural heart disease upstream of traditional echocardiogram laboratories.^6^ Single-probe devices are now found in practice and are being studied for their ability to reduce costs and shorten time to rule in (or out) preliminary diagnoses,^7^ there remain significant concerns related to image quality^8^ and interpretation^9,10^ by non-experts.

While cardiac ultrasound image quality from single-probe handheld devices has improved over the last few years, there is no information about how often cardiac imaging from modern devices is interpretable for cardiac structure or function. Recognizing the widespread use of these newer multi-purpose devices, this study tests the hypothesis that interpretable cardiac imaging can be obtained using single-probe handheld ultrasound devices. The study aims are to quantify cardiac image quality, describe associations with sex and BMI, and determine the frequency of uninterpretable imaging from these devices.

## Methods

We conducted a single-centre, prospective study of focused cardiac ultrasound (FoCUS) imaging from single-probe handheld devices (*Graphical Abstract*). Imaging was acquired by registered cardiac sonographers in The Tufts Cardiovascular Imaging and Hemodynamic Laboratory where TTEs are regularly performed on both hospitalized and ambulatory patients. All patients referred for clinically indicated comprehensive TTE were eligible for inclusion. The study protocol was approved by the Institutional Review Board, and all participants provided written informed consent.

### Imaging protocol

Default cardiac imaging settings for each device were used. Following standard TTE imaging, FoCUS imaging using a single-probe handheld device was performed. In standard fashion cardiac sonographers saved optimized imaging of limited standardized views. FoCUS views included parasternal long-axis (PLAX), parasternal short-axis at the level of the aortic valve (PSAX AoV), apical 2-chamber (A2C), and apical 4-chamber (A4C) views. The number of acquisitions of each imaging view was not pre-specified.

### Image quality assessment

Leveraging prior work^2,11,12^ that focused on a cardiac-specific imaging probes, we employed a simplified grading schema that incorporated image brightness, resolution and region of interest to determine image quality.^5-7^ Video samples for each patient (range 1–5) were manually imported into an image viewer (see Supplement  *S1***)** that allowed cine-loops to be viewed and scored in a blinded manner. Cine-loops were graded with a summary score on a scale of 1 (uninterpretable) to 5 (diagnostic-quality imaging) by two sonographers blinded to imaging device type (***Figure 1***). Sonographers involved in the quality grading attended a calibration session of exemplar handheld imaging representing grades 1–5 prior to the grading procedure. These example images were available for reference throughout the grading exercise. Each cine-loop was assigned an imaging quality grade according to the following criteria: 1 (image quality too poor to permit meaningful interpretation), 2 (image quality poor, but some cardiac structure/function can be seen), 3 (suboptimal image quality, but basic image interpretation was possible), 4 (image interpretation possible but limited), and 5 (good image quality allowing meaningful interpretation). Anatomically relevant features varied based on imaging plane (e.g. PLAX vs. A4C). Cine loops with an average IQ score <2 were classified as uninterpretable; those with an average IQ score ≥2 were classified as interpretable. Cine-loops were considered good quality if average IQ score ≥ 4.

**Figure 1:**
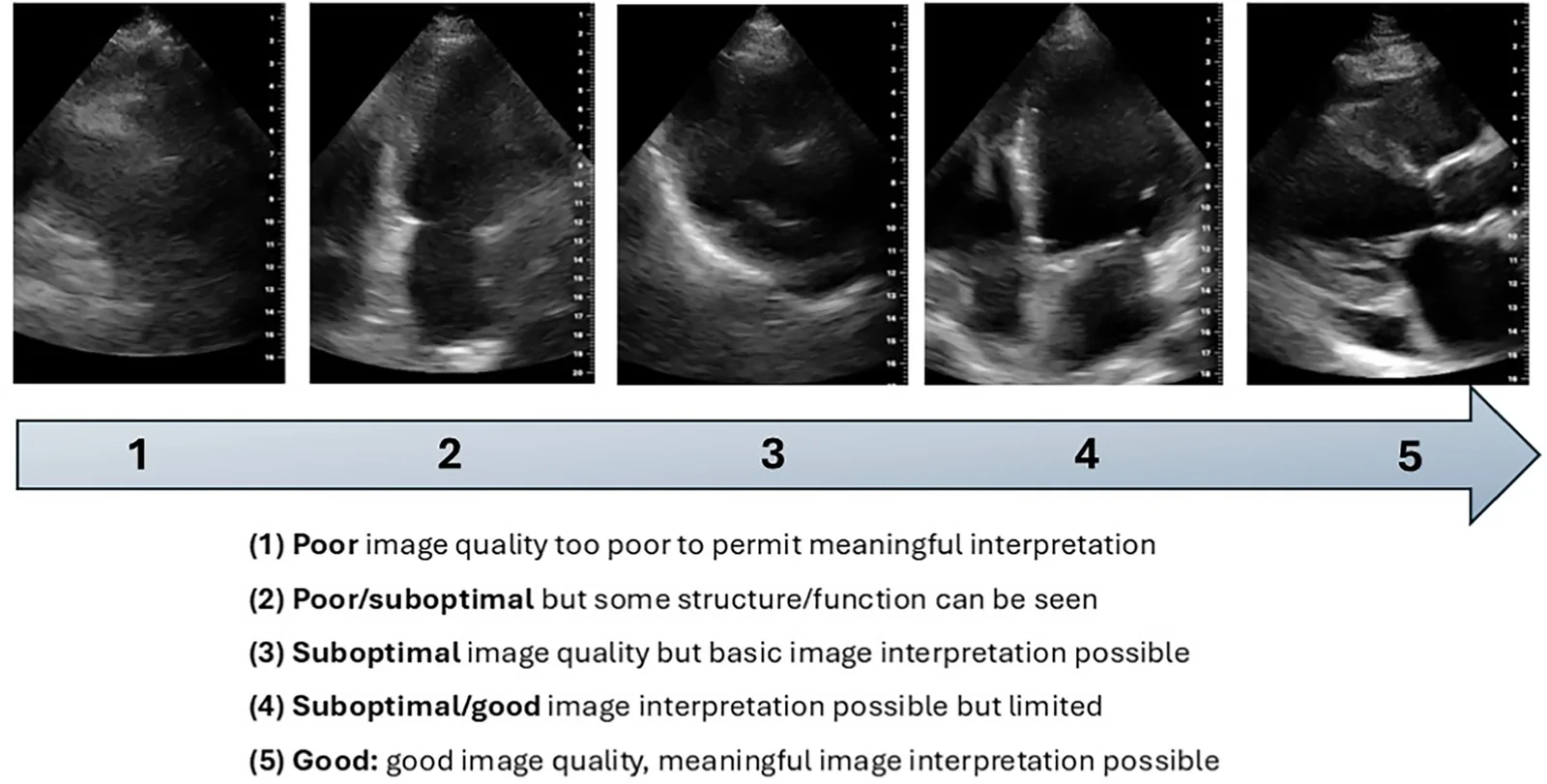
Sample representative still frame images of various image quality grades. Image quality was assessed at the video level as a single numeric grade. Videos were graded for image quality by two registered sonographers and the average grade was used for analysis.

### Clinical variables

Study participants’ electronic medical records were reviewed and clinical data were extracted from the electronic medical record. Clinical variables including age, sex, body mass index and left ventricular ejection fraction, and comorbidities including hypertension, hyperlipidemia, congestive heart failure, and diabetes were extracted.

### Statistical analysis

The primary outcome of interest was video-level image quality. Average image quality was calculated as the mean of the two sonographer ratings for each cine loop. Because this averaged outcome could take nine possible values from 1.0 to 5.0 in 0.5-point increments, it was treated as a quasi-continuous outcome. Associations of average image quality with view type, sex, and BMI were evaluated using linear mixed-effects models with a patient-level random intercept to account for multiple cine loops within patients. Model-based effect estimates and 95% confidence intervals were reported. Binary interpretability outcomes were analysed using logistic generalized estimating equations with patient as the clustering unit. All models used an exchangeable working correlation structure and empirical sandwich estimators for robust standard error estimation. Since images-per-patient were not pre-specified for this report, patient-level analyses are presented in a descriptive manner only.

This study evaluated a fixed cohort of 518 diagnostic images collected across 206 unique patients, so we conducted a post-hoc power and minimal detectable effect analysis accounting for patient-level clustering, looking at the primary outcome of image interpretability. With an average cluster size of 2.5 images per patient (518/206) and an empirical intraclass correlation coefficient (ICC) of 0.8. The design effect was 2.2, which yielded an effective sample size of 234 observations. Based on this effective sample size, a two-tailed alpha=0.05 and 80% statistical power, the study had 80% power to detect a minimum absolute risk difference of 16.3% in image interpretability between sexes and a 17.4% risk difference in image interpretability comparing the highest BMI group to the other two groups.

Inter-rater agreement for the ordinal image quality ratings was assessed using weighted Cohen's kappa. To account for clustering of multiple cine loops within patients, 95% confidence intervals were obtained using a patient-level block bootstrap with 1000 resamples. Continuous variables were summarized using means and standard deviations or medians and Q1-Q3, as appropriate, and categorical variables using counts and percentages. All tests were two-sided.

## Results

Imaging from three single-probe handheld imaging devices (Vave®, Butterfly IQ+®, Butterfly IQ3®) representing 518 cine-loops from 206 patients was assessed (***Table 1***). The average number of cine loops per patient was 2.51 (SD 0.58, ***Figure 2***). 100 patients (49%) were scanned with the Butterfly IQ + probe, 50 (24%) were scanned with the Butterfly IQ3 probe, and 56 (27%) patients were scanned with the Vave probe. The mean patient age was 72 (SD 11.1) years, and the study cohort consisted of 110 male patients (53.4%) and 96 female patients (46.6%). The cohort was predominantly White (156/206, 75.7%) and non-Hispanic or Latino (199/206, 96.6%). The mean patient height was 168.8 cm (SD 11.66), and mean weight was 79.9 kg (SD 20.15) with a mean BMI of 27.8 (SD 5.36). Mean LVEF was 55.4% (SD 12.4). Common comorbidities included hypertension (168/206, 81.6%) and hyperlipidaemia (159/206, 77.2%). Congestive heart failure (68/206, 33%) and diabetes (49/206, 23.8%) were less common among study participants.

**Figure 2:**
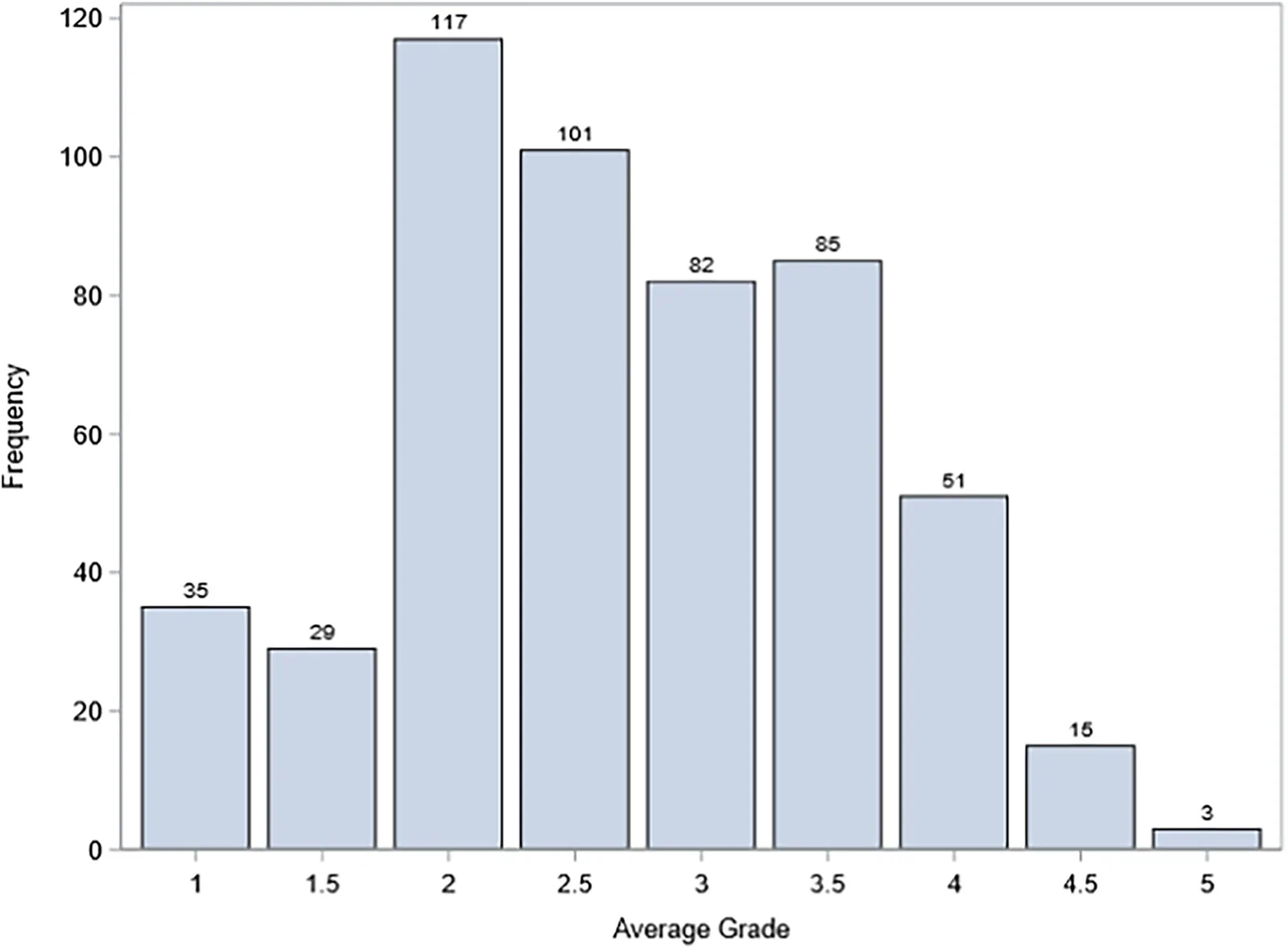
Average image quality score across the study cohort. Each video was graded by two registered sonographers blinded to device type. The average score was used for analysis.

**Table 1:** Baseline characteristics of the study population stratified by handheld ultrasound device type

	Type			
	Total (N = 206)	ButterflyIQ + (N = 100)	ButterflyIQ3 (N = 50)	Vave (N = 56)
**Age, mean (SD)**	71.9 (11.1)	74.3 (8.5)	63.8 (14.4)	74.9 (7.8)
**Sex**, n (%)				
Male	110 (53.4%)	52 (52.0%)	32 (64.0%)	26 (46.4%)
Female	96 (46.6%)	48 (48.0%)	18 (36.0%)	30 (53.6%)
**Race/ethnicity**, n (%)				
** American Indian/Alaskan Native**	1 (0.5%)	1 (1.0%)	0 (0.0%)	0 (0.0%)
** Asian**	26 (12.6%)	12 (12.0%)	6 (12.0%)	8 (14.3%)
** Black**	16 (7.8%)	8 (8.0%)	2 (4.0%)	6 (10.7%)
** White**	156 (75.7%)	74 (74.0%)	40 (80.0%)	42 (75.0%)
** Other**	3 (1.5%)	1 (1.0%)	2 (4.0%)	0 (0.0%)
** Unknown**	4 (1.9%)	4 (4.0%)	0 (0.0%)	0 (0.0%)
**Ethnicity**, n (%)				
** Hispanic of Latino**	3 (1.5%)	1 (1.0%)	2 (4.0%)	0 (0.0%)
** Not Hispanic or Latino**	199 (96.6%)	95 (95.0%)	48 (96.0%)	56 (100.0%)
** Unknown**	4 (1.9%)	4 (4.0%)	0 (0.0%)	0 (0.0%)
**Height, mean (SD)**	168.8 (11.7)	168.3 (11.9)	172.5 (11.0)	166.5 (11.3)
**Weight, mean (SD)**	79.9 (20.2)	80.7 (22.1)	84.6 (18.0)	74.2 (16.9)
**BMI, mean (SD)**	27.8 (5.4)	28.0 (5.7)	28.7 (4.6)	26.6 (5.3)
**Ejection fraction, mean (SD)**	55.4 (12.4)	56.1 (12.0)	53.2 (12.6)	55.9 (12.8)
**Hypertension**, n (%)	168 (81.6%)	94 (94.0%)	33 (66.0%)	41 (73.2%)
**Hyperlipidemia**, n (%)	159 (77.2%)	84 (84.0%)	29 (58.0%)	46 (82.1%)
**Congestive HF**, n (%)	68 (33.0%)	38 (38.0%)	12 (24.0%)	18 (32.1%)
**Diabetes**, n (%)	49 (23.8%)	28 (28.0%)	11 (22.0%)	10 (17.9%)

Patients were recruited in the echocardiogram laboratory at the time of clinically indicated transthoracic echocardiography. Race/ethnicity is self-identified.

### Video-level image quality

The median average IQ score was 2.5 (IQR 1.5). Four hundred fifty-four (88%) of the cine-loops from 206 patients were classified as interpretable (average image quality ≥ 2) while 64 (12%) were classified as uninterpretable (average IQ < 2) (***Figure 2***). Inter-rater agreement was moderate, with a weighted kappa of 0.50 (95% CI 0.45–0.55) (***Figure 3***). Image quality was determined as good (average IQ score ≥ 4) by both sonographers for 62/518 (12%) of cine-loop images. Average IQ grade did differ across the view types (*P* < 0.01) but did not differ across sex (mean difference: −0.04; CI: −0.27 to 0.18; *P* = 0.67), ***Figures 4*** and ***5***. Overall, PLAX had the highest average IQ grade. PSAX (AoV) and Apical 2 and Apical 4 view types had significantly lower average IQ grades (PSAX (AoV): mean difference: −0.36; CI: −0.46 to −0.25; *P* < 0.001; Apical 2: mean difference: −0.26; CI: −0.50 to −0.03; *P* = 0.03; Apical 4: mean difference: −0.12; CI: −0.33 to 0.10; *P* = 0.29), There was a trend towards lower image quality with increasing BMI tertiles (***Figures 6****A* and ***6****B* (BMI 16–25, median IQ score 3.0 (IQR 1.5); BMI 25–29, median IQ score 3.0 (IQR 1.5); BMI > 29, median IQ score 2.5 (IQR 1.0), *P* < 0.001). Compared to the BMI > 29 category, the BMI 16–25 category had an average IQ score 0.47 higher (CI: 0.21 to 0.74), and the BMI 25–29 category had an average IQ score 0.47 higher (CI: 0.20 to 0.74). The proportion of uninterpretable images did not differ across sex (*P* = 0.69; OR 0.87; CI: 0.43 to 1.75), or BMI category (overall *P* = 0.26; pairwise: lowest category compared to highest OR: 1.86; 95% CI: 0.83 to 4.17; middle category compared to highest OR: 1.96; 95% CI: 0.78 to 4.93).

**Figure 3:**
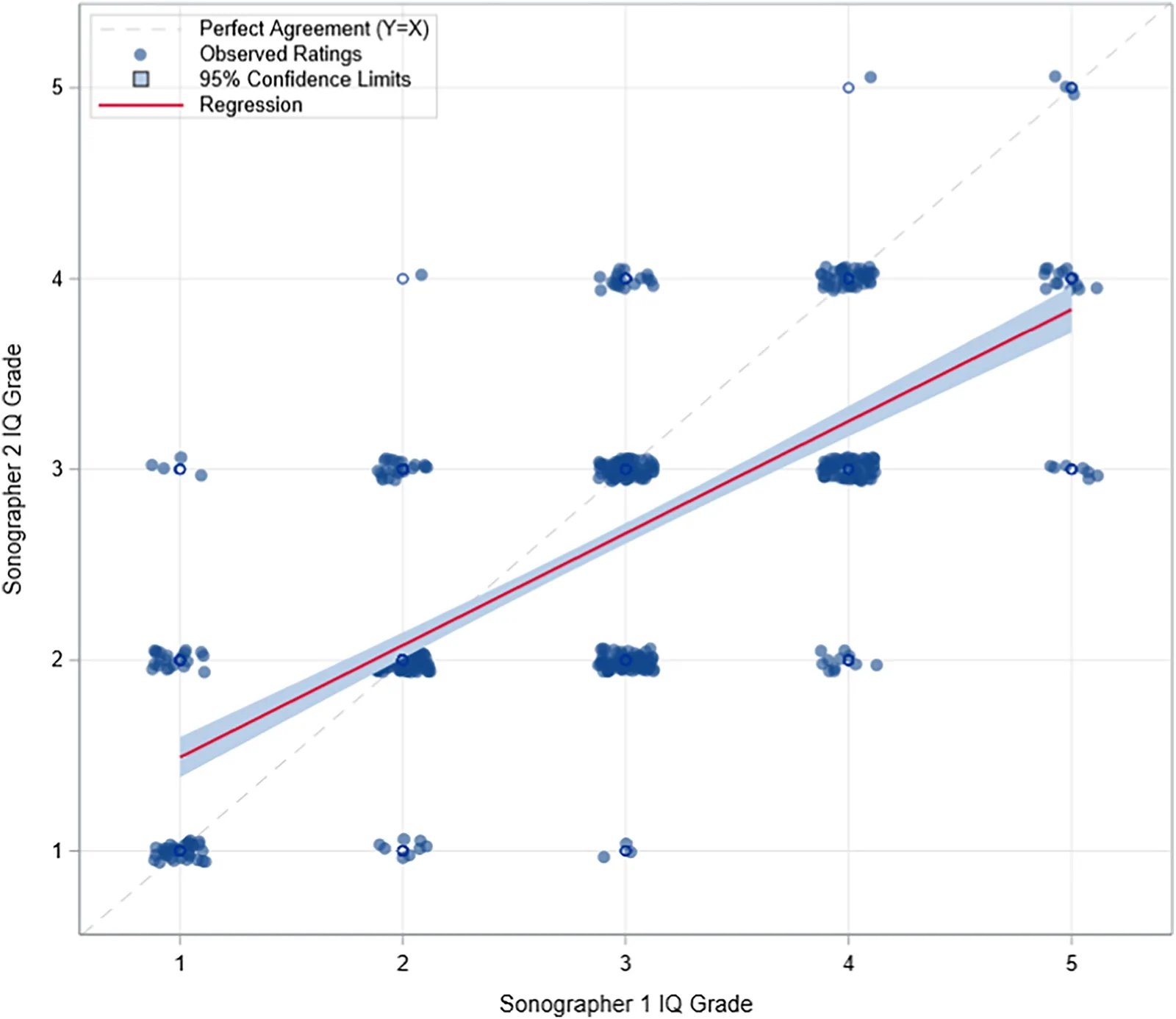
Distribution of paired sonographer image quality ratings. Videos were assigned an ordinal Image Quality score that ranged 1–5 by two registered sonographers blinded to device type. IQ grades were assigned as follows: 1 (image quality too poor to permit meaningful interpretation), 2 (image quality poor but some structure/function can be seen), 3 (sub-optimal image quality though basic image interpretation possible), 4 (image interpretation possible but limited), and 5 (good image quality with meaningful image interpretation possible). Correlation between sonographer IQ grades was 0.60 and weighted kappa was 0.50 (95% CI 0.45–0.55).

**Figure 4:**
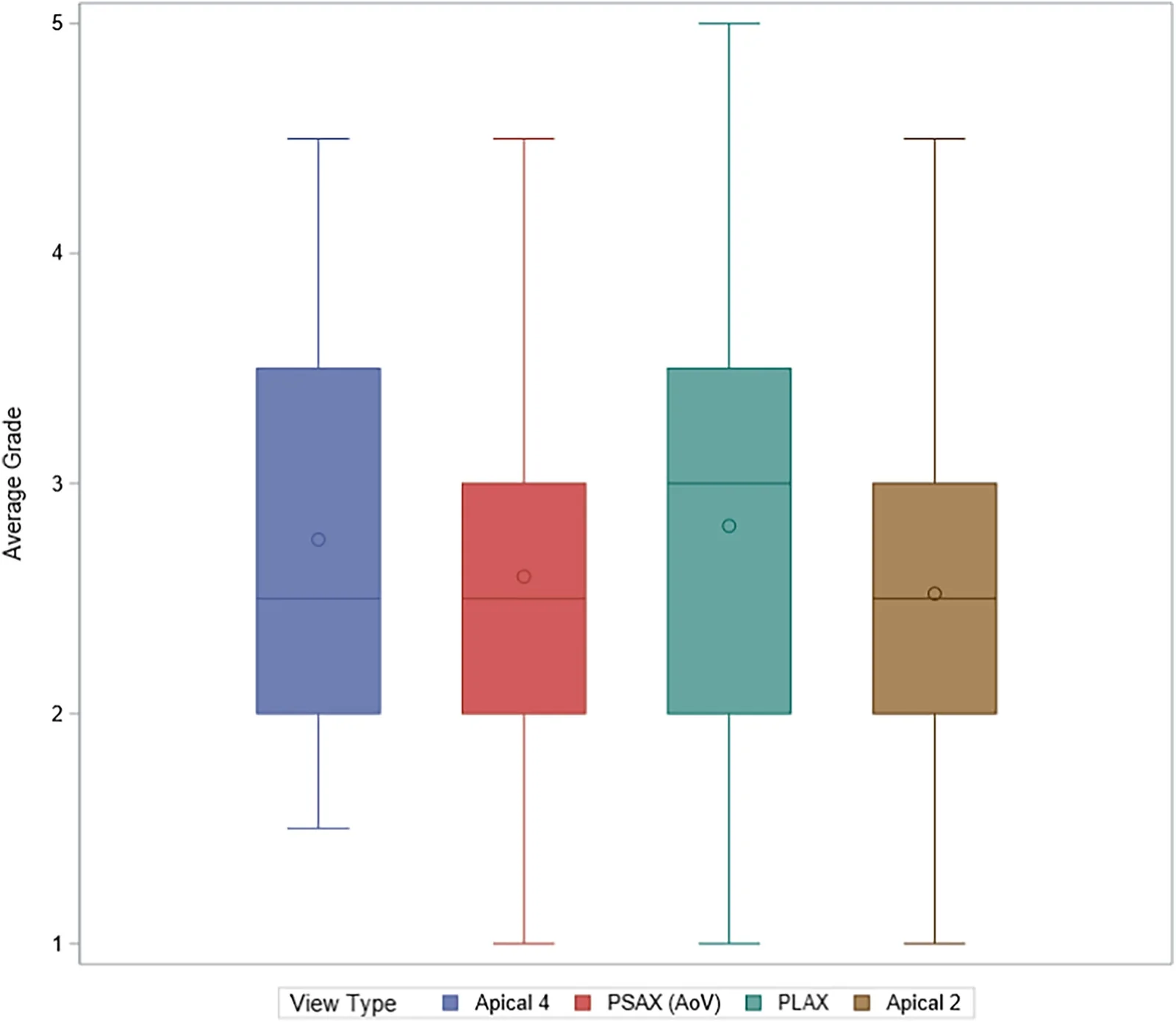
Image quality across view type. Apical 4 is apical 4 chamber view, PSAX (AoV) is parasternal short axis at the level of the aortic valve. PLAX is parasternal long axis, and Apical 2 is apical 2 chamber imaging.

**Figure 5:**
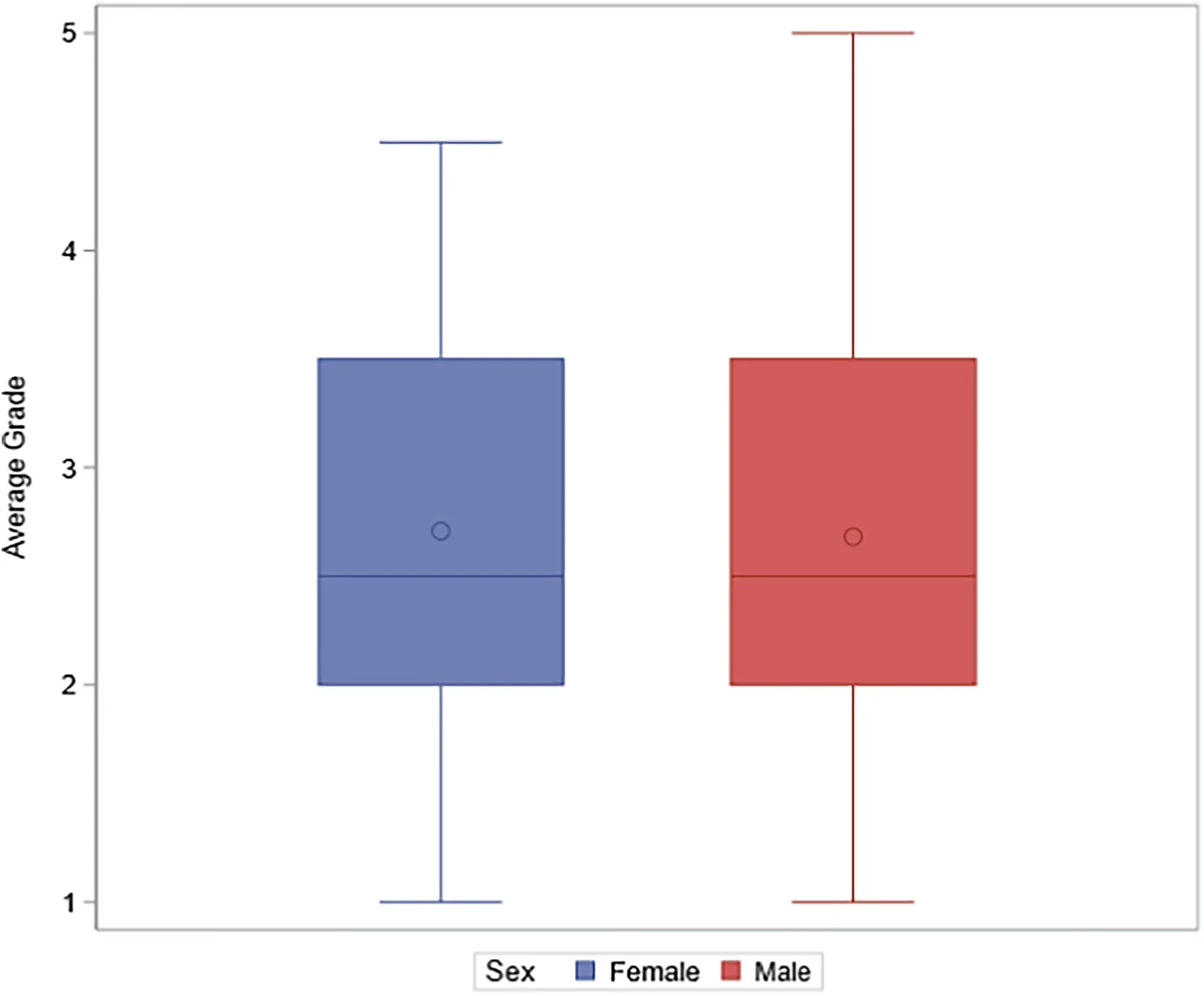
Video level analysis of image quality according to sex.

**Figure 6:**
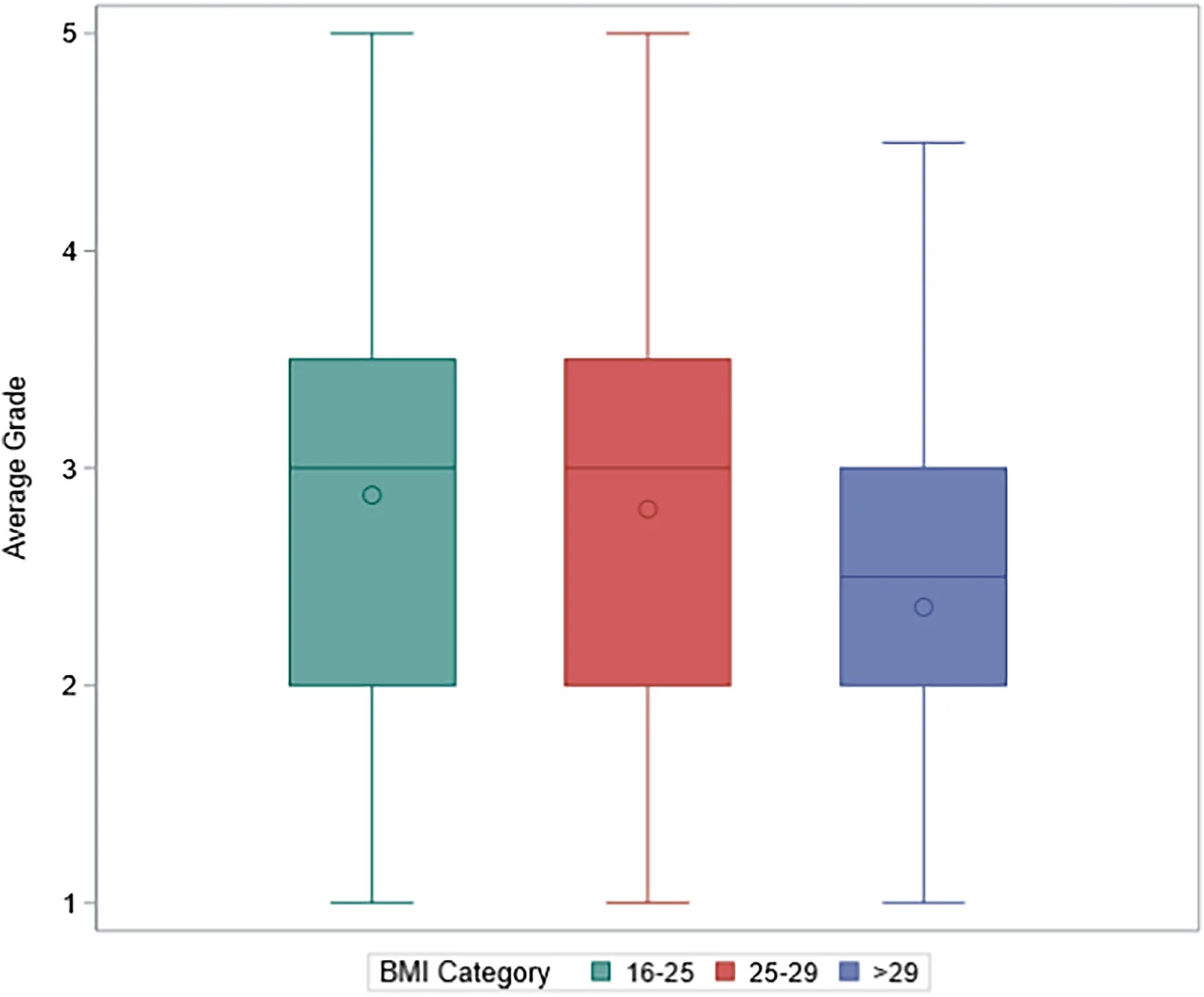
Video level analysis of image quality according to BMI.

### Patient-level image quality

At least one sampled cine-loop with an average image quality ≥ 2 was obtained for 187 (91%) patients. The median best (highest) patient level image quality was 3 (2.5–3.5). 42 (20%) patients had ≥ 1 good quality video (average IQ score ≥ 4). Nineteen (9%) patients had only uninterpretable imaging (all imaging with average IQ score < 2). The highest image quality was most commonly found in the PLAX view (N = 121; 59%) followed by the PSAX (AoV) view (N = 37; 18%). Patient-level descriptive analysis revealed that the median highest average IQ grade was 3 (IQR 1.5) for men and 3 (IQR 1.25) for women, ***Figure 7***. Image quality across BMI categories is shown in ***Figure 8*** (BMI 16–25, highest median IQ score 3.0 (IQR 1.25); BMI 25–29, highest median IQ score 3.0 (IQR 1.0); BMI > 29, highest median IQ score 2.5 (IQR 1.0). The proportion of patients without interpretable imaging (best average IQ < 2) was 5% (95% CI 1–15%), 8% (95% CI 3–15%), 16% (95% CI 7–29%), for Vave, Butterfly IQ+, Butterfly IQ3, respectively. The proportion of patients without interpretable imaging was 12% (95% CI 6–19%) for men and 6%, (95% CI 2–13%) for women.

**Figure 7:**
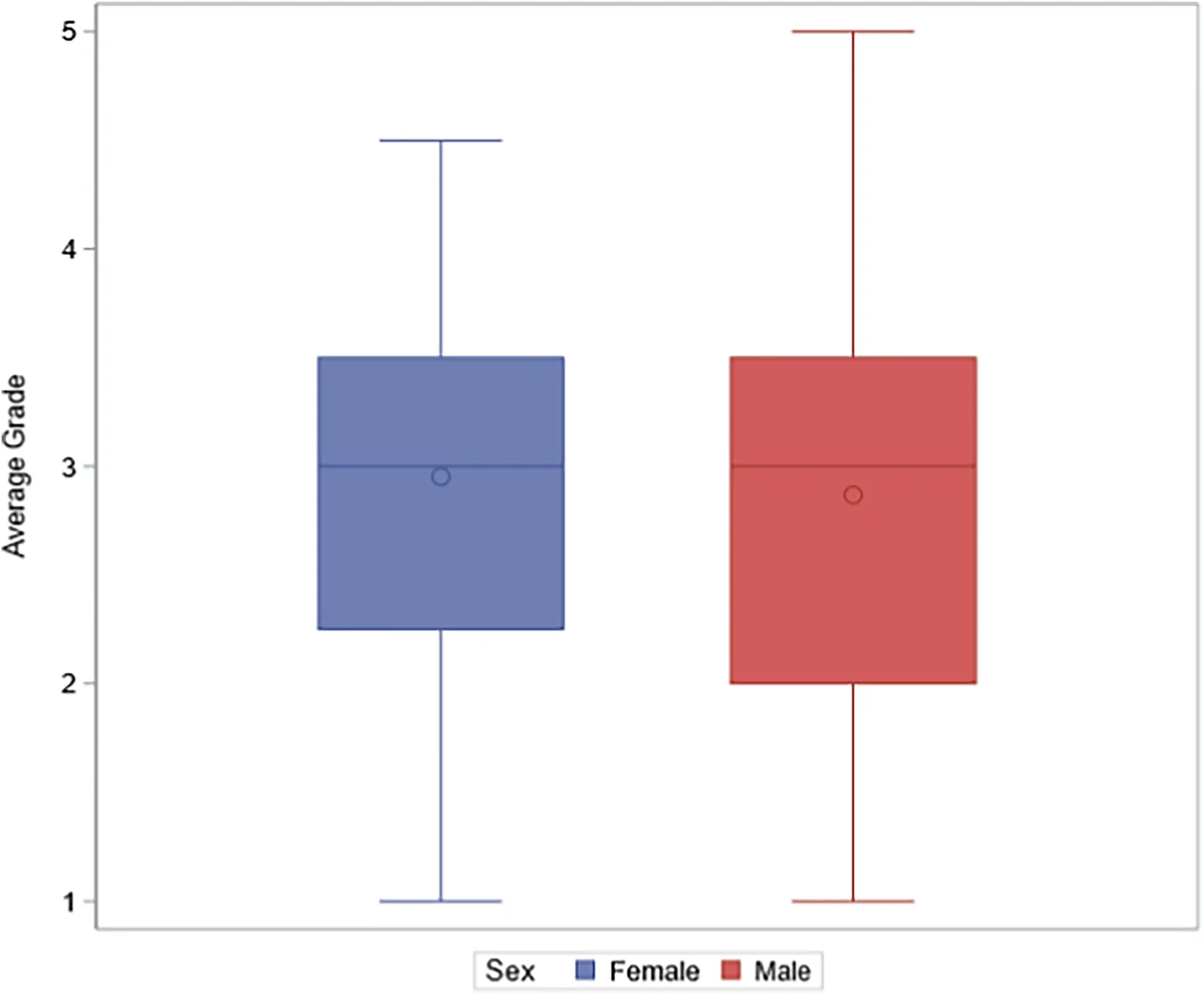
Patient-level analysis of image quality according to sex. The ‘best’ imaging for a patient was used for this analysis.

**Figure 8:**
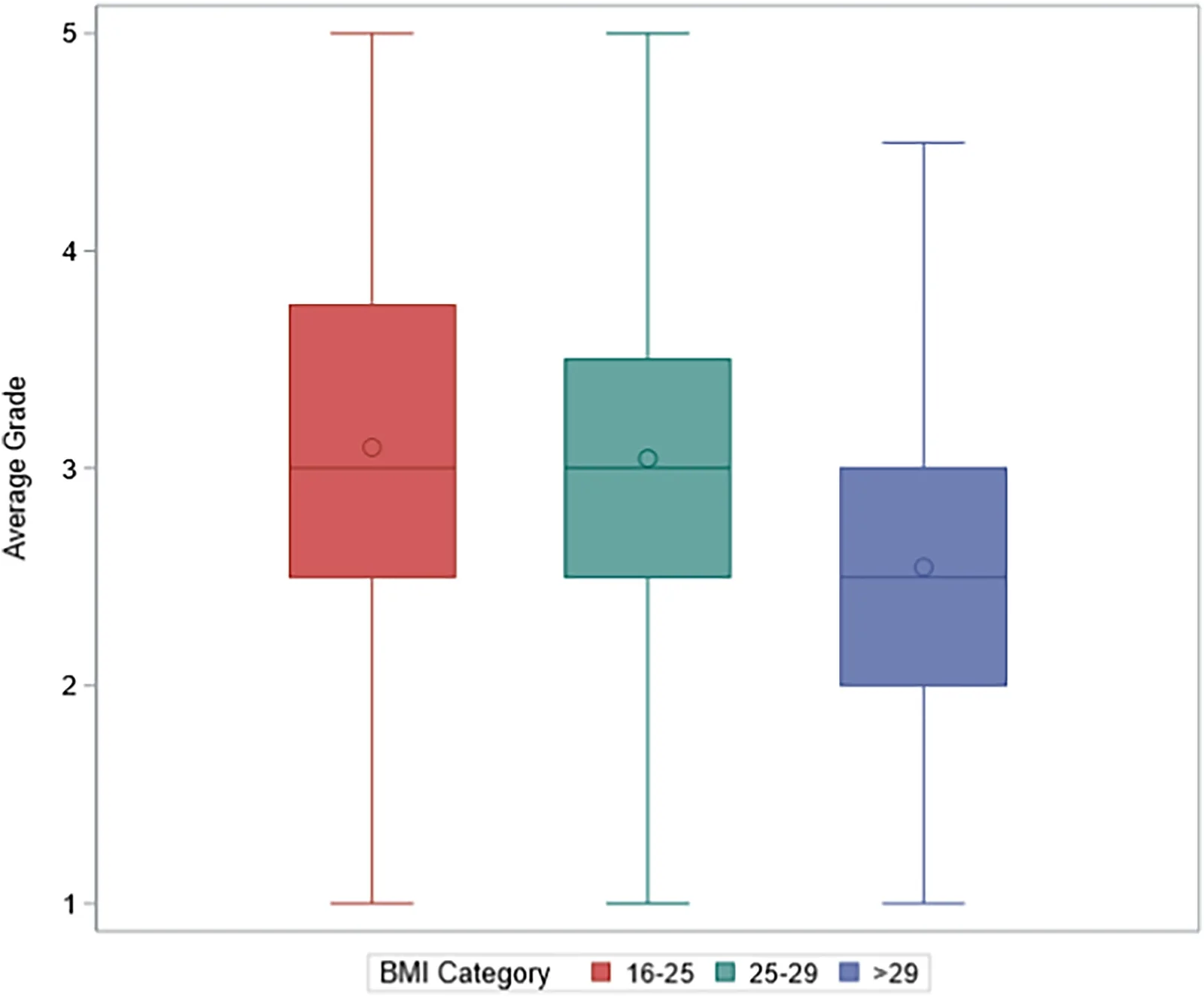
Patient-level analysis of image quality by BMI. The ‘best’ imaging for a patient was used for this analysis.

## Discussion

The primary finding from this study is that interpretable cardiac imaging can be obtained from most patients with single-probe handheld ultrasound devices; however, good image quality is rare and imaging is uninterpretable for a significant minority of patients. While these probes hold significant potential to improve detection of cardiac pathology, there remains a critical need to understand, for any given patient, whether underlying image quality can enable meaningful interpretation. Here, imaging from the PLAX window showed the highest average image quality and there was no difference in image quality across sex; however, lower image quality was associated with higher BMIs. This report provides an expert-acquisition benchmark for future studies evaluating handheld FoCUS performed by nonexpert users and demonstrates the potential (and limitations) of cardiac acquisitions from modern single-probe handheld ultrasound devices.

Single-probe devices represent an important advance in ultrasound technology because they can image across anatomic regions at various depths. This feature has led to substantial interest among generalist and emergency/critical care providers, and these devices are increasingly integrated into teaching and clinical care pathways. While there are emerging examples of how these devices can enable cardiac imaging and interpretation by non-experts^13^ and might improve clinical decision-making and decrease resource utilization,^7^ image quality and concerns about accurate interpretation persist among cardiac imaging experts. This study shows that single-probe ultrasound devices yield interpretable cardiac imaging from most patients, while imaging was uninterpretable for either structure or function for 9% of patients (12% of sampled cine-loops in this study). These results from expert imagers in a controlled setting likely represent a performance ceiling for these devices, and future work will focus on the quality of imaging done by non-experts in various clinical settings.

Quality assessment for handheld imaging is essential to ensure accuracy of clinical information obtained and subsequent decision-making. Various factors contribute to differences in image quality including operator experience^14^ and hardware limitations^15^ driven by small handheld device size. In this study lower image quality was associated with higher BMI, an observation that raises concerns about routine image interpretation for patients with obesity. Overall, while the average study was interpretable for basic cardiac structure and function, there are important limitations to imaging with these devices that should be recognized. First, while certain structures are well suited to simple views (valve structure and movement, myocardial wall thickness and function, aorta dimensions), there are important disease states that are not effectively captured by limited imaging from these probes (e.g. regurgitant lesions that require colour Doppler imaging, stenotic lesions that require continuous wave Doppler imaging, and complex phenotypes like cardiac amyloid or multiparametric valve disease staging). While machine-learning classifiers might improve detection of some of these conditions,^16^ the impact on clinical care and outcomes requires additional study and more work is needed to establish an evidence base for which probe/imaging protocol/setting/condition will improve patient outcomes.

We present a simplified IQ scoring system focused on the key imaging domains that are relevant for handheld cardiac imaging interpretation: basic cardiac structure and function. The scoring system demonstrated moderate inter-rater agreement between imaging experts and offers two advantages over other grading tools in the literature.^17–19^ First, the score used here offers a summary assessment of the ‘interpretability’ of a single video acquisition (as opposed to an assessment of multi-video imaging protocols). This single-video focus more closely aligns with how generalists or critical care providers use handheld cardiac imaging (i.e. to rule-in or -out specific diagnoses). Second, our group is optimizing machine learning-based disease detectors for handheld cardiac ultrasound specifically considering absention methods^20^ that can learn when to ignore low-quality videos to improve performance and trust when classifiers are deployed into clinical care. Adoption of a standardized schema for assessing FoCUS image quality from single-probe devices will be essential for consistent quality assessment to establish trust and optimize appropriate interpretation of imaging from these devices.

This study has limitations that should be recognized. Since patients were not randomized to device assignment, device comparisons cannot establish comparative performance. Differences in the scanned populations may contribute to apparent performance differences (e.g. higher average BMI in the group scanned with Butterfly IQ3). These results may not extend to other device types or other settings where imaging done by non-experts is likely to be lower quality. Additional techniques for image optimization (brightness, gain settings) might further enhance image quality but require an additional level of skill for non-experts to master. In this study default ‘cardiac’ settings were used. Since analyses here were based on sampled cine loops, they should not necessarily be interpreted as the probability of successfully acquiring interpretable imaging. Additionally, since patient level summaries depend at least partly on the number of available images, they are presented in a descriptive manner. Lastly, inter-rater agreement between the two sonographers was moderate.

## Conclusions

Interpretable cardiac imaging from single-probe devices can be obtained for most patients; however, imaging from a significant minority of patients is uninterpretable for either cardiac structure or function. A simple image quality score can reliably be assigned to FoCUS cine-loops to help identify scans that are appropriate (or inappropriate) for interpretation.

## Supplementary Material

qyag172_Supplementary_Data

## Data Availability

The data underlying this article cannot be shared publicly due to the privacy of individuals that participated in the study. The data may be shared on reasonable request to the corresponding author.

## Lead author biography

Anna Beatrice Grimaldo is a third-year Internal Medicine resident at Tufts Medical Center. She earned her medical degree from Mayo Clinic Alix School of Medicine in Rochester, MN and her Masters in the Science of Healthcare Delivery from Arizona State University.

## References

[1] d’Arcy JL, Coffey S, Loudon MA, Kennedy A, Pearson-Stuttard J, Birks J et al Large-scale community echocardiographic screening reveals a major burden of undiagnosed valvular heart disease in older people: the OxVALVE population cohort study. Eur Heart J 2016;37:3515–22. 10.1093/eurheartj/ehw229 PMC5216199 27354049

[2] Kirkpatrick JN, Grimm R, Johri AM, Kimura BJ, Kort S, Labovitz AJ et al Recommendations for echocardiography laboratories participating in cardiac point of care cardiac ultrasound (POCUS) and critical care echocardiography training: report from the American society of echocardiography. J Am Soc Echocardiogr 2020;33:409–22.e4. 10.1016/j.echo.2020.01.008 32122742

[3] Douflé G, Teijeiro-Paradis R, Morales-Castro D, Urner M, Goffi A, Dragoi L et al Point-of-care ultrasound: a case series of potential pitfalls. CASE 2022;6:284–92. 10.1016/j.case.2022.05.002 PMC9399537 36036054

[4] Spencer KT, Kimura BJ, Korcarz CE, Pellikka PA, Rahko PS, Siegel RJ. Focused cardiac ultrasound: recommendations from the American society of echocardiography. J Am Soc Echocardiogr 2013;26:567–81. 10.1016/j.echo.2013.04.001 23711341

[5] Brener MI, Chuang ML, Nishimura R, Hamid NB, Snyder C, Thompson JB et al Population prevalence of valvular heart disease in the United States. JACC 2026;87:3227–39. 10.1016/j.jacc.2026.02.5137 42300818

[6] Chang A, Strom JB, Liu K. AI-Assisted Point-of-Care ultrasound networks—considerations for rural health care delivery. JAMA Cardiol 2025;10:643. 10.1001/jamacardio.2025.0829 40305012

[7] Maganti K, Chen C, Jamthikar AD, Parikh P, Yanamala N, Sengupta PP. Cardiopulmonary point-of-care ultrasonography for hospitalist management of undifferentiated dyspnea. JAMA Netw Open 2025;8:e2530677. 10.1001/jamanetworkopen.2025.30677 PMC12413653 40911308

[8] Adabifirouzjaei F, Kimura BJ, Khazai B, DeMaria AN. Handheld ultrasonography in cardiovascular medicine, updates and controversies. Prog Cardiovasc Dis 2022;74:70–9. 10.1016/j.pcad.2022.10.012 36404443

[9] Cardim N, Dalen H, Voigt J-U, Ionescu A, Price S, Neskovic AN et al The use of handheld ultrasound devices: a position statement of the European association of cardiovascular imaging (2018 update). Eur Heart J Cardiovasc Imaging 2019;20:245–52. 10.1093/ehjci/jey145 30351358

[10] Chamsi-Pasha MA, Sengupta PP, Zoghbi WA. Handheld echocardiography: current state and future perspectives. Circulation 2017;136:2178–88. 10.1161/CIRCULATIONAHA.117.026622 29180495

[11] Cawthorn TR, Nickel C, O’Reilly M, Kafka H, Tam JW, Jackson LC et al Development and evaluation of methodologies for teaching focused cardiac ultrasound skills to medical students. J Am Soc Echocardiogr 2014;27:302–9. 10.1016/j.echo.2013.12.006 24433979

[12] Spencer FA, Lopes LC, Kennedy SA, Guyatt G. Systematic review of percutaneous closure versus medical therapy in patients with cryptogenic stroke and patent foramen ovale. BMJ Open 2014;4:e004282. 10.1136/bmjopen-2013-004282 PMC3948581 24607561

[13] Trost B, Rodrigues L, Ong C, Dezellus A, Goldberg YH, Bouchat M et al Artificial intelligence empowers novice users to acquire diagnostic-quality echocardiography. JACC Adv 2025;4:102005. 10.1016/j.jacadv.2025.102005 PMC12308015 40700992

[14] Jenkins S, Alabed S, Swift A, Marques G, Ryding A, Sawh C et al Diagnostic accuracy of handheld cardiac ultrasound device for assessment of left ventricular structure and function: systematic review and meta-analysis. Heart 2021;107:1826–34. 10.1136/heartjnl-2021-319561 PMC8562308 34362772

[15] Athreya S, Radhachandran A, Ivezić V, Sant VR, Arnold CW, Speier W. Enhancing ultrasound image quality across disease domains: application of cycle-consistent generative adversarial network and perceptual loss. JMIR Biomed Eng 2024;9:e58911. 10.2196/58911 PMC11688586 39689310

[16] Karmiy S, Huang Z, Velury D, Mai E, Li J, Dehn MM et al Machine learning-enabled screening for aortic stenosis with handheld ultrasound. Eur Hear J—Imaging Methods Pract 2025;3:qyaf051. 10.1093/ehjimp/qyaf051 PMC12089772 40395285

[17] Ultrasound guidelines: emergency, point-of-care and clinical ultrasound guidelines in medicine. Ann Emerg Med. 2017;69:e27–54. 10.1016/j.annemergmed.2016.08.457 28442101

[18] Adamson R, Morris AE, Sun Woan J, Ma IWY, Schnobrich D, Soni NJ. Development of a focused cardiac ultrasound image acquisition assessment tool. ATS Sch 2020;1:260–77. 10.34197/ats-scholar.2020-0002OC PMC8043312 33870293

[19] DeMasi S, Taylor LA, Weltler A, Wiggins JC, Wayman J, Wang C et al Novel quality assessment methodology in focused cardiac ultrasound. Acad Emerg Med 2022;29:1261–3. 10.1111/acem.14562 PMC9804740 35842913

[20] Kompa B, Snoek J, Beam AL. Second opinion needed: communicating uncertainty in medical machine learning. NPJ Digit Med 2021;4:4. 10.1038/s41746-020-00367-3 PMC7785732 33402680

