# Supplementary material for: Assessment of cardiac image quality from single-probe handheld ultrasound devices
Source: Eur Heart J Imaging Methods Pract. 2026 Oct 6;4(4):qyag172. doi: 10.1093/ehjimp/qyag172 (PMC13685631; doi:10.1093/ehjimp/qyag172)
Supplement: Supplementary file 1 [file qyag172_supplementary_data.docx]

Supplement

S1. IQ Grading Software.
